# Potential Role of Selenoenzymes and Antioxidant Metabolism in relation to Autism Etiology and Pathology

**DOI:** 10.1155/2014/164938

**Published:** 2014-03-05

**Authors:** Laura J. Raymond, Richard C. Deth, Nicholas V. C. Ralston

**Affiliations:** ^1^Energy & Environmental Research Center, University of North Dakota, 15 North 23rd Street, Stop 9018, Grand Forks, ND 58202, USA; ^2^Department of Pharmaceutical Sciences, Northeastern University, Boston, MA 02115, USA

## Abstract

Autism and autism spectrum disorders (ASDs) are behaviorally defined, but the biochemical pathogenesis of the underlying disease process remains uncharacterized. Studies indicate that antioxidant status is diminished in autistic subjects, suggesting its pathology is associated with augmented production of oxidative species and/or compromised antioxidant metabolism. This suggests ASD may result from defects in the metabolism of cellular antioxidants which maintain intracellular redox status by quenching reactive oxygen species (ROS). Selenium-dependent enzymes (selenoenzymes) are important in maintaining intercellular reducing conditions, particularly in the brain. Selenoenzymes are a family of ~25 genetically unique proteins, several of which have roles in preventing and reversing oxidative damage in brain and endocrine tissues. Since the brain's high rate of oxygen consumption is accompanied by high ROS production, selenoenzyme activities are particularly important in this tissue. Because selenoenzymes can be irreversibly inhibited by many electrophiles, exposure to these organic and inorganic agents can diminish selenoenzyme-dependent antioxidant functions. This can impair brain development, particularly via the adverse influence of oxidative stress on epigenetic regulation. Here we review the physiological roles of selenoproteins in relation to potential biochemical mechanisms of ASD etiology and pathology.

## 1. Introduction

The causes of autism and autism spectrum disorder (collectively, ASD) remain unknown, in part because of complex behavioral phenotypes and the likelihood that multiple genetic and environmental factors contribute to its etiology [1–3]. In the absence of biochemical tests for ASD, the diagnosis is based solely on clinical assessment of behavioral criteria that define deficits in social interaction, impairments in verbal and nonverbal receptive/expression, speech, and hyperfocused repetitive behaviors. The pathophysiology of ASD is primarily expressed in the neurologic, immunologic, and gastrointestinal (GI) systems and affects four times as many boys as girls [4–6]. Regression, with loss of previously acquired skills, can also interrupt apparently normal development. Children with severe autism can exhibit mental retardation, and autistic children have an elevated rate of seizure disorders [7].

Although ASD was previously thought to be rare, the number of persons receiving treatment for ASD has increased substantially during the past several decades and continues to increase. A recent US government report estimated the prevalence of ASD increased by 78% from 2002 to 2008 [8]. In 2011-2012, a prevalence of 20 per 1000 was reported for school aged children [9]. However, a portion of ASD's increasing incidence may reflect changes in diagnostic practice and the broadening of diagnostic criteria [10]. Other studies indicate a diagnostic shift or substitution may also have contributed to the rise in diagnosis, whereby the increase in autism diagnoses corresponds with declines in the usage of other diagnostic categories [11, 12]. Based on a meta-analysis of ASD studies, McDonald and Paul [13] concluded that it does not seem possible to assess whether or how much of the observed increases in cumulative incidence are real, although the number of individuals identified as having ASD has increased dramatically.

Until reliably accurate differential diagnoses are achieved, it is difficult to attain the goal of defining the biochemical and physiological lesions that initiate and/or perpetuate the dysfunctions of autism. However, one pathological mechanism present in many children with ASD involves defects in the control of oxidative damage. Distinctions in the nature of these perturbations in redox control may provide insight for identifying biochemically defined patient subgroups that may respond to specific therapeutic interventions. This paper focuses on potential associations between genetic and acquired defects in control of oxidative damage, particularly those that impinge upon selenium- (Se-)dependent enzymes (selenoenzymes). Se physiology is a vital process in the brain and neuroendocrine system, [25, 26] and, because of the high reactivity and low abundance of Se in these tissues, its vulnerability to inhibition by a variety of toxicants is markedly enhanced. However, its potential role in the pathophysiology of ASD remains largely unexplored.

## 2. Oxidative Stress in ASD

Chemically reactive oxygen-derived products like peroxide radicals (^∙^O_2_^2−^), hydrogen peroxide (H_2_O_2_), superoxide–anion (O_2_^−^), singlet oxygen (^1^O_2_), and hydroxyl radicals (^∙^OH), are products of ongoing aerobic metabolism via mitochondrial oxidative phosphorylation [27]. If not intercepted and detoxified, reactive oxygen species (ROS) are capable of chemically damaging all forms of cellular macromolecules. To avoid these consequences, numerous ROS-detoxifying reactions enable cells to maintain redox equilibrium and metabolic homeostasis. Thus, oxidative stress is a condition where the level of ROS production exceeds antioxidant capacity.

Increased oxidative stress has been observed in children with ASD [28–30]. Blood collected from autistic children shows low concentrations of membrane polyunsaturated lipids, higher phospholipase A_2_, and loss of the normal asymmetry of membrane lipoproteins, which may indicate increased oxidative damage [13, 31]. Levels of endogenous and exogenous antioxidant capacity are commonly reduced in ASD. Glutathione (GSH) is the primary intracellular antioxidant, and the ratio of its reduced (GSH) and oxidized (GSSG) forms (GSH/GSSG) provides a useful index of redox status. As shown in Table 1, numerous studies have reported significantly lower plasma levels of GSH and, in some cases, lower GSH/GSSG levels. Low GSH levels are associated with oxidative stress, increased inflammation, impaired immune response, and a decreased ability to detoxify environmental contaminants. Autistic children have been reported to be increasingly susceptible to recurrent infections, neuroinflammation, gastroinflammation, and impaired antioxidant and detoxification capacity. Diminished glutathione peroxidase (GPx), superoxide dismutase, and catalase enzyme activities have been associated with ASD, as well as low cysteine, Se, zinc (Zn), and Vitamins C, E, and A [32], although these associations are not consistently observed.

Accumulation of oxidized glutathione (GS-SG) in plasma is a strong indication of intracellular oxidative stress, as cells export the GS-SG to maintain redox equilibrium. James and coworkers [14] were first to report that plasma levels of cysteine, GSH, and the GSH/GS-SG ratio were significantly decreased in autistic children. In that study, total GSH levels were decreased, and GS-SG was increased, resulting in a threefold reduction in the redox ratio of GSH/GS-SG. Cysteine, the rate-limiting amino acid for GSH synthesis, was significantly decreased relative to controls in over 65% of the autistic children tested. The finding of lower-plasma GSH has since been replicated by other research groups [17–20, 33], suggesting this is a prevalent feature of ASD. A recent meta-analysis finds that children with ASD have decreased blood GSH (27%), GPx activity (18%), and methionine (13%) and increased concentrations of GS-SG (45%) relative to nonautistic children [34]. In addition, levels of NADPH and NADH, which reflect redox status and help maintain GSH in its reduced state, were found to be significantly lower in autistic children [32]. Several studies have reported a decrease in the level of GSH in postmortem brain samples from ASD subjects, associated with a decrease in the GSH/GS-SG ratio and increased levels of oxidative stress biomarkers [35, 36]. In addition, activities of several GSH-related enzymes, including the selenoenzyme GPx, were lower in cerebellums of ASD subjects [37].

The folate and Vitamin B_12_-dependent enzyme methionine synthase (MS) is inhibited under oxidative stress conditions, resulting in a decrease in all methylation reactions, including DNA methylation [38]. The basis for MS inhibition is oxidation of its Vitamin B_12_ (cobalamin) cofactor, which is considered the most readily oxidized biomolecule, making it an ideal sensor of cellular redox status [39, 40]. Lower MS activity inhibits methylation by lowering the ratio of the methyl donor S-adenosylmethionine (SAM) to the methylation inhibitor S-adenosylhomocysteine (SAH), exerting a global dampening effect on >200 methylation reactions. Arguably, the most important among these are methylation of DNA and histones, which combine to exert epigenetic regulation over gene expression. As noted above, a decrease in SAM/SAH has been documented in plasma of ASD subjects in association with a decrease in GSH/GS-SG, reflecting the reciprocal relationship between oxidative stress and methylation. In neuronal cells methionine synthase activity is stimulated by growth factors and dopamine but inhibited by neurodevelopmental toxins, including mercury (Hg) [41]. Methionine synthase mRNA levels are significantly decreased in postmortem brains of autistic subjects, indicative of a deficit in methylation capacity secondary to oxidative stress [42].

Elevated urinary levels of 8-isoprostane-F_2*α*_ (8-iso-PGF_2*α*_) and malondialdehyde (MDA), oxidative stress biomarkers, have also been noted in children with autism [28, 30]. A bimodal distribution of 8-iso-PGF_2*α*_ was reported, with the majority of autistic subjects showing moderate increases in isoprostane levels, while a smaller group of autistic children showed dramatic increases. Levels of urinary 8-hydroxy-2-deoxyguanosine (8-OHdG), a major product of DNA oxidation, were also measured but did not reach statistical significance, although they indicated a trend toward increasing concentrations in children with autism [30]. No significant correlations were noted between the levels of these biomarkers and vitamin intake, dietary supplements, medicine, medical disorders, or history of regression. Therefore, these results suggest that lipid peroxidation is increased in autistic children and that certain autistic children have much greater oxidative stress than others.

## 3. Selenium-Dependent Antioxidant Metabolism and ASD

**Figure 1:**
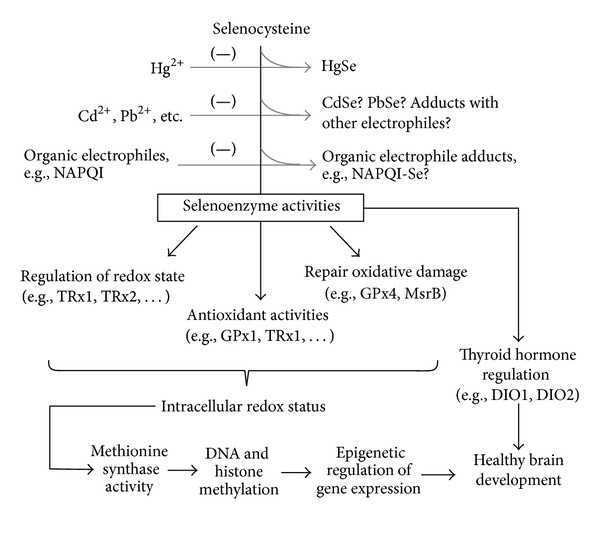
Selenoprotein synthesis and activities are sensitive to elemental and organic electrophiles. High exposures to soft electrophiles may additively impair redox regulation and thyroid hormone production, disrupting epigenetic regulation and normal brain development.

Selenoproteins are essential for brain development, redox control, and preventing and reversing oxidative damage in the brain and neuroendocrine tissues (Figure 1, Table 2). Therefore, control of intracellular oxidative tone and findings of increased oxidative damage in children with ASD may be indicative of disruptions of selenoenzyme activities. The molecular forms of Se most common in foods are the amino acids selenocysteine (Sec) and selenomethionine (SeMet), although traces of water-soluble inorganic forms (e.g., selenate and selenite) can also be present in food and drinking water. For both the organic and inorganic Se forms, biochemical utilization in selenoenzymes is initiated through the common intermediate hydrogen selenide (H_2_Se). Therefore, all ingested (and endogenous) forms of Se must be degraded to inorganic selenide before Se can be used for synthesis of Sec, the physiological active form of Se. Although proteins with SeMet contain Se, they are not considered selenoproteins because SeMet is nonspecifically incorporated into proteins as if it were Met. The nonspecific insertion of SeMet or Met is directed by AUG codons and no significant distinctions in the biochemical functions have been observed. This is in contrast to Sec, which is the catalytically active primary amino acid present in all selenoproteins [43] and is responsible for the principal functions of these enzymes (Table 2). In contrast to other amino acids, Sec is not recycled for reincorporation into new proteins but is, instead, degraded to release inorganic Se which can be utilized for resynthesis of Sec.

While Cys, the analogous sulfur amino acid, is inserted at UGU/UGC codons, the insertion of Sec is in response to UGA which is otherwise the “opal” stop codon for other proteins [44–46]. The selenoprotein mRNAs include a distinct Sec insertion sequence (SECIS) stem-loop structure in their untranslated 3′ region of the mRNA. This is recognized by specific SECIS binding proteins which function together with several transacting factors as well as a unique tRNA with an anticodon complementary to UGA to initiate de novo Sec synthesis. The tRNA is aminoacylated with serine prior to biosynthesis of Sec which is inserted into the protein's primary structure. In mRNA of most selenoproteins, the UGA of the Sec insertion codon is followed by a second or terminal UGA, which is then read as the stop codon.

Since the discovery of these genetically unique proteins, their enzyme activities have become increasingly well defined. Associations between compromised Se and ASD have been reported including low Se levels in red blood cells [47]. Since blood Se is less prone to contamination and more indicative of tissue selenoenzyme activities, it is considered to be a more reliable index than hair. Indeed, hair Se is variously reported as being increased [48], decreased [49], or unchanged [50] in subjects with ASD.

When considering the developmental roles of selenoproteins, as well as their involvement in redox control and protection from oxidative stress, the potential for selenoenzyme dysregulation in relation to the pathologies associated with ASD appears worthy of investigation. The three main families of characterized selenoproteins—iodothyronine deiodinases (DIO), thioredoxin reductases (TRx), and GPx—have critical roles in thyroid function, fetal development, hormone metabolism, and oxidative stress detoxification, particularly in endocrine and brain tissues. Although ~25 selenoproteins are known, only members of the major selenoenzyme/selenoprotein families with potential relevance to autism etiology and pathology are discussed below.

### 3.1. Roles of Selenoenzymes in Thyroid Hormone Regulation

Selenoenzymes regulate thyroid synthesis and metabolic functions contributing to thyroid hormone biosynthesis, antioxidant defense, redox control of thyrocytes, and thyroid hormone metabolism. Thyroid hormones have important roles in regulating many key biochemical reactions, especially protein synthesis and enzymatic activity, accompanied by an increase in basal metabolic rate. Thyroid hormone regulates several processes that are associated with brain differentiation, including dendrite and axon growth, synaptogenesis, neuronal migration, and myelination [51]. Disruption of thyroid hormone production during early child development leads to permanent deficiency in intelligence and sensorimotor functions [52], and it is hypothesized that Se deficiency may be responsible for the initiation of autoimmune thyroid disorders [53]. Interestingly, thyroid hormone increases plasma Se levels, as well as the levels of selenoenzymes such as DIO [54]. This relationship is logical, since increased metabolic activity places a higher demand on antioxidant resources.

The central nervous system is very sensitive to thyroid hormone supply during growth and development. The selenoprotein family of DIOs (DIO1, 2, and 3) is involved in the formation and regulation of the active thyroid hormone, triiodothyronine (T_3_). More than 80% of T_3_ in the brain is derived from intracellular deiodination of T_4_ by DIO2 [55, 56]. Since circulating T_3_ does not readily gain access to intracellular nuclear receptors [57], DIO2 provides an important regulatory function in the brain and central nervous system (CNS). During early childhood, T_3_ bound to nuclear receptors is entirely dependent on its local production from T_4_ via this selenoprotein.

### 3.2. Roles of TRx in Redox Regulation

**Figure 2:**
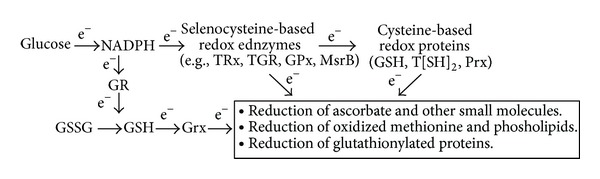
Selenoenzymes are central to providing antioxidant electrons to accomplish reduction of molecules in a number of biochemical processes. NADPH = nicotinamide adenine dinucleotide phosphate; GR = glutathione reductase; T(SH)_2_ = reduced thioredoxin; GSH = reduced glutathione; TRx = thioredoxin reductase; GSSG = oxidized glutathione; TGR = thioredoxin-glutathione reductase; GPx = glutathione peroxidase; MsrB = methionine sulfoxide reductase; Prx = peroxiredoxin; Grx = glutaredoxin; e^−^ = electron. Levels of plasma GSH, erythrocyte NADH, and NADPH are notably reduced (*P* < 0.001) in children with autism [16, 32].

The three distinct forms of TRx—TRx1, TRx2, and TRx3 (collectively, TRx)—are important in controlling redox state within major compartments of the cell. While TRx1 (cytosolic) and TRx2 (mitochondrial) restore oxidized thioredoxin (T[S-S]) to its reduced (T[SH]_2_) form and are responsible for reducing a variety of other essential antioxidant molecules [63] including Vitamin C [62]. Thioredoxin is a ubiquitous 12 kDa protein that employs vicinal cysteines (CXXC motif) and becomes oxidized to intramolecular disulfides T(S-S) during reduction of other molecules (Figure 2). Its action is essential for countering oxidative damage in the cytosol of aerobic organisms from bacteria to humans [68]. Since T(SH)_2_ is a central regulator of cellular redox status that is required for the redox-regulated function of transcription factors and hormonally regulated nuclear receptors, it is critical in DNA production, gene expression, cell survival, and embryogenesis. Thus TRx maintains T(SH)_2_ levels to enable basic processes and regulate multiple metabolic events. The antioxidant functions of TRx occur because they directly facilitate reduction of oxidized proteins through cysteine-thiol-disulfide exchange, forming an oxidized disulfide T(S-S) in the process. TRx is also directly involved in prevention and repair of damage caused by H_2_O_2_-based oxidative stress. Because intracellular reduction of selenite is required for de novo synthesis of Sec selenoproteins, TRx clearly has a central role in all Se physiology. It is assumed that Se's pivotal role in TRx explains why targeted disruption of the TRx1 [69] and T(SH)_2_ [70] genes are embryonically lethal.

Additionally, investigations have shown that TRx1 synthesized without its penultimate Sec is an apoptosis initiator (GRIM-12) [71]. The only difference between the truncated GRIM-12 and full-length TRx1 is the absence of the final two amino acids, Sec and glycine. Truncation occurs when the codon UGA is interpreted as a stop codon instead of a signal for Sec insertion during times of Se deficiency or when its Sec is selectively derivatized. The Sec-deficient form of TRx1, GRIM-12, is a notably powerful apoptosis initiator that rapidly induces cell death [71].

### 3.3. Roles of GPx in Redox Regulation

GPx (five genetically distinct forms, GPx1–4 and GPx6) are selenoenzymes involved in antioxidant defense and redox regulation and modulation. GPx provide protection against oxidative damage and aid in the maintenance of membrane integrity by using GSH as a cofactor to catalyze reduction of hydrogen peroxide, forming oxidized glutathione (GS-SG) in the process. Thyroid hormone synthesis requires a continuous production of high concentrations of H_2_O_2_, which appears to be its rate-limiting step [72–74]. Therefore, since the thyrocyte is continually exposed to potentially toxic concentrations of H_2_O_2_ and lipid hydroperoxides, appropriate antioxidant defense systems are essential to control excess oxidative stress. Three of the five GPx are expressed in thyrocytes and thyroid tissue [75–77]. Studies indicate a distinct regulation of expression, secretion, and function of these selenoproteins for controlling thyrocyte growth, differentiation, and function [76–83]. When Se intake is adequate, the intracellular GPx and TRx systems protect the thyrocyte from peroxides; however, in Se deficiency, the thyrocyte's apoptotic response to H_2_O_2_ is increased [84]. Furthermore, in iodine deficiency, where hyperstimulation of the thyroid-stimulating hormone (TSH) receptor signals increased H_2_O_2_ production, GPx production is also stimulated, thus upregulating antioxidant protection. By virtue of its ability to increase basal metabolism, thyroid hormone increases oxygen utilization, thereby increasing the demand for antioxidant.

GPx4 reduces hydroperoxides of membrane phospholipid fatty acids and has particular relevance for autism. Along with TRx1, TRx2, DIO2, DIO3, and selenoprotein P (SelP), GPx4 is considered an essential selenoprotein whose levels are preserved in brain and endocrine tissues during Se deficiency. Suppression of neuronal GPx4 expression resulted in a selective loss of parvalbumin-expressing GABAergic interneurons [85] that are essential for dopamine-dependent regulation during attention [86] and has been linked to attention deficit hyperactivity disorder (ADHD) [87, 88]. Earlier studies also showed that expression of these interneurons was inhibited when glutathione synthesis was impaired, indicating a critical role for redox status in establishing the capacity for attention [89, 90].

### 3.4. Selenoenzyme Metabolism and Physiology

In addition to the TRx and GPx selenoenzyme families, further selenoproteins have recently been implicated in processes known to be involved in neurodegenerative diseases, including protein folding, degradation of misfolded membrane proteins, and control of cellular calcium homoeostasis [91]. Cerebral Se deficiency is associated with neurological disorders such as seizures and ataxia [92, 93], consistent with a restriction in the development of inhibitory interneurons. Knockout of selenoprotein synthesis in neurons specifically interfered with development of parvalbumin-expressing GABAergic interneurons, and knockout of GPx4 produced a similar deficit, indicating that these neurons have a particular requirement for Se [85]. Thus impaired selenoprotein synthesis or loss of their activities could contribute to the neurocognitive dysfunction and seizure activity in ASD.

The cerebral cortex, hippocampus, cerebellum, and olfactory bulb express the highest numbers of selenoproteins [94]. The brain and endocrine tissues are preferentially supplied with Se, predominantly through directed distribution and cellular uptake of SelP. Although other selenoproteins uniformly incorporate only a single Sec per molecule, SelP uniquely contains 10 Sec per molecule. Studies with SelP-deficient mice indicate that moderate reductions of brain Se content will impair brain function [95, 96].

Not only is SelP important for Sec transport, but it also appears to have a vital role in neurogenesis. A study of its brain distribution found a remarkably higher concentration in ependymal cells, which are found at the ventricle surface [97]. Ependymal cells are a source of neural stem cells which are produced upon asymmetric cell division and give rise to neuronal, astrocyte and oligodendrocyte lineages in the subependymal region [98]. Neurotrophic growth factors stimulate mitosis of these precursor cells [99] and provide an important source of postnatal neurons. SelP is taken up by neurons via apolipoprotein E receptor 2 (ApoER2), which is localized to synapses, and ApoER2 knockout mice show a decrease in synapse density as well as a decrease in the number of dendritic spines [100, 101].

All brain selenoenzymes are affected by loss of Se in the absence of SelP, but the DIOs, TRxs, and GPxs are the selenoproteins considered to have the most critical roles in the brain and endocrine system. Therefore, loss or compromise of their functions [96] would have dramatic effects on maturation of the neuroendocrine system. The unusual capacity of the brain and endocrine tissues (e.g., pituitary, testes) to retain their selenoenzyme activities during prolonged or even multigenerational deficiency states [43] indicates that their redox regulation effects are important in these tissues. The low GSH level of neurons (~0.2 mM) provides a unique opportunity for redox signaling as a mechanism for epigenetic control. Neurotrophic factors stimulate cysteine uptake and increase both GSH/GS-SG and SAM/SAH in association with a significant increase in DNA methylation [102]. However, Se-dependent redox regulation is more vulnerable to soft electrophiles, positively charged chemical species which bind to selenoproteins with exceptionally high affinity, as discussed below.

## 4. Genetic Influences and Metabolic Disturbances in ASD

Observations from family studies suggest that ASD has a strong genetic component [103–105], although the failure to identify genetic factors affecting more than a small proportion of ASD cases suggests that multiple etiologies may be responsible for the pathologies and neurobehavioral features of the disorder [106–117]. Moreover, genetic factors may increase the probability of oxidative damage and diminish the body's ability to detoxify ROS and free radicals. Interactions between genetic and environmental factors may potentiate increased oxidative stress in autistic children.

Genetic risk of autism may be related to a differential sensitivity to environmental factors. Using a strict definition of autism, a recent study found a 58% concordance rate for monozygotic male twins and 60% for females and 21% and 27% for male and female dizygotic twins, respectively [118]. Using the broader definition of ASD, monozygotic concordance increased to 77% and 50% for males and females, while dizygotic concordance was 31% and 36%, respectively. These rates are substantially lower than earlier estimates, and the authors concluded that environmental factors are more important than genetic factors, although genetic factors clearly play an important role. Moreover, no individual genetic cause of autism has been identified to account for more than 1%-2% of cases and, with the exception of Rett syndrome, there is no current evidence that ASD is linked to any specific genetic or nongenetic disorder. However, there is evidence suggesting that epigenetic factors and exposures to environmental modifiers may contribute to variable expression of autism-related traits [42]. Variants of major effect genes and numerous common variants with smaller effect genes have been identified in individuals with ASD and related conditions. These genetic variances are providing insights to common pathways and metabolic disturbances affected in ASD, particularly genes involved in oxidative stress and detoxification pathways [15, 119, 120].

Polymorphisms of genes involved in glutathione metabolism, including genes for GPx and glutathione S-transferase (GST), have been reportedly associated with ASD. GPx1 is the predominant and most abundant isoenzyme of GPx and plays an integral role in reducing oxidative stress by catalyzing the reduction of potentially harmful peroxides. Ming et al. [121] found significant disequilibrium in the overall transmission of a sequence polymorphism of GPx1 in ASD. Williams et al. [122] showed that the GSTP1-313A allele may be acting as a teratogenic allele, contributing to the phenotype of the affected child. GST proteins conjugate and detoxify products of oxidative stress and conjugate toxins that produce oxidative stress. By assessing genotypes of mothers and maternal grandparents, it was shown that the GSTP1A haplotype was significantly more frequently transmitted to mothers of individuals with ASD, suggesting that it may be acting in mothers during pregnancy to contribute to the phenotype of autism during fetal development [122].

James et al. [15] examined the frequency of several single nucleotide polymorphisms (SNPs) capable of affecting redox and methylation pathways in autistic subjects. They found significant differences in allele frequencies for the reduced folate carrier (RFC 80G>A), transcobalamin II (TCN2 776 G>C), methylenetetrahydrofolate reductase (MTHFR) 677 C>T and 1298 (A>C), catechol-O-methyltransferase (COMT 472 G>A), and GST M1 between autistic and control cohorts. These differences were associated with abnormal metabolite levels, suggesting that individuals with genetic vulnerability affecting redox and methylation capacity may be linked to a higher risk for autism. Any deficit in the function of selenoproteins could synergize with these genetic risk factors.

DNA copy number variants (CNVs) represent a major category of genetic risk for ASD and are implicated in approximately 10% of cases [123, 124]. Several of the genes likely affected by homozygous deletions are regulated by neuronal activity, and the expression of these genes can change in response to neuronal stimulation. Synapses mature partially as a function of experience-dependent neuronal activity, so disruption of those genes by mutation or copy number variation may alter the process of synaptic development. DNA methylation status is associated with the occurrence of CNVs [125], raising the possibility that impaired methylation capacity could contribute to increased CNVs in ASD.

## 5. Epigenetic Disturbances in ASD

Epigenetic regulation utilizes covalent modifications such as DNA methylation and the addition/removal of various chemical moieties to histone tails (collectively known as epigenetic marks) to provide stable, transgenerational changes in gene expression without alteration of the underlying nucleotide sequence [126, 127]. Epigenetic marks are dynamic and highly sensitive to cellular changes [128]. Thus, normal physiologic changes in the cellular environment, such as levels of growth factors, hormones, and neurotransmitters, as well as xenobiotic exposure, can translate into modifications in gene expression mediated by epigenetic regulation. Xenobiotic exposures affecting epigenetic status can, therefore, not only produce lifelong consequences, but their effects can be transmitted through germline cells to affect multiple succeeding generations [126, 128].

As noted above, MS exerts powerful control over all methylation reactions via its influence over the SAM/SAH ratio, and MS inhibition by oxidative stress will cause both a decrease in SAM and an increase in SAH, while reducing conditions will have the opposite effect. A twofold increase in MS activity induced by IGF-1 is associated with a twofold increase in global DNA methylation, while inhibition of MS activity by ethanol is associated with a large decrease [41]. Thus, xenobiotics affecting redox status can exert an epigenetic influence.

Beyond its direct epigenetic regulation of gene transcription, DNA methylation also regulates the activity of repetitive transposable elements dispersed throughout the human genome. Transposable elements comprise about 45% of the genome, and their earlier description as “junk DNA” has recently been revised in recognition of their ability to modulate gene transcription, mRNA splicing, micro-RNA formation, and other processes [129]. Reflecting their viral origin, retrotransposons such as the LINE-1 (long interspersed nuclear element-1) family are suppressed by methylation but can replicate and transpose to new locations, especially during early development and especially when methylation is suppressed. Based upon its impressive quantitative contribution to the genome, methylation of LINE-1 has been used as a surrogate for global DNA methylation [130], and factors regulating MS activity affect LINE-1 methylation [131]. LINE-1 retrotransposition is reported to occur at a higher rate in brain than to other tissues [132], and a higher rate was observed in Rett syndrome subjects carrying mutations in the methylated DNA binding protein MeCP2 [130]. Although more studies are needed to clarify their specific contribution, transposable elements such as LINE-1 are poised to provide a global genomic influence during development, so agents affecting their methylation state are likely to disrupt this process.

Oxidative stress and decreased methylation capacity are common in autism and abnormal epigenetic regulation may link the metabolic abnormalities to disruptions in brain development. Other comorbid features of autism, such as autoimmunity and gastrointestinal (GI) dysfunction [133, 134], may reflect similar manifestations of abnormal epigenetic regulation. A genome-wide comparison of DNA methylation in monozygotic twins discordant for autism found numerous differentially methylated regions associated with ASD, and the extent of these differences were correlated with severity of autistic trait scores [135].

From conception to maturation, human development is a highly orchestrated expression of epigenetic regulation, so it is not surprising that genetic and environmental factors adversely affecting oxidative tone and methylation status can contribute to developmental disorders. The exceptionally dynamic redox-dependent epigenetic regulation in the brain increases its vulnerability to neurodevelopmental disorders. Autism is a prominent feature of Rett, Angelman, Prader-Willi, and Fragile-X syndromes, each of which has been linked to interruption of methylation-dependent regulation [136–138]. Therefore, environmental exposures affecting redox and methylation status could reasonably result in neurodevelopment disorders such as ASD.

## 6. ASD in relation to Exposures to Potentially Neurotoxic Agents

Certain soft electrophiles are known to be neurotoxic at high exposures, presumably due to their effects on sulfur metabolism. Although these electrophilic species are highly interactive with cellular nucleophiles such as thiols, the Se of Sec is by far the strongest intracellular nucleophile. Therefore, selenoproteins are very vulnerable to enzyme inhibition by binding to neurotoxic electrophiles. Their toxic concentrations are generally miniscule in relation to sulfur, toxic levels generally equal or exceed the normal tissue concentrations of Se. Therefore, because of their high reactivity and low molar abundance, selenoenzymes are highly vulnerable to selective inhibition by high concentrations of electrophiles such as Hg. Soft electrophiles such as Hg have larger ionic radii and a more dispersed surface charge, making them more reactive with soft nucleophiles such as the Se of Sec at the active sites of enzymes.

Only certain electrophiles are notably neurotoxic. Those that bind and potentially sequester Se would cooperatively diminish the biological availability of Se for performance of its necessary physiological roles (Figure 1). For example, a multitude of electrophilic agents are naturally present in food in small amounts and the Se-sequestering effects of each would usually be minor. However, their additive effects on selenoenzyme synthesis and function could be detrimental in individuals with compromised Se status or metabolism.

Likewise, additional exposures to electrophiles are encountered in the form of environmental contaminants, such as toxic metals, pesticides, herbicides, and others. In addition, soft electrophiles are present as the active ingredients in many pharmaceuticals and food preservatives, while still others are produced during the degradation of these products. Therefore, instead of examining relationships between ASD incidence and exposures to individual agents, it may be more informative to examine ASD incidence in relation to aggregate exposures to these soft electrophiles and/or their effect on selenoenzyme activity in vulnerable individuals.

### 6.1. Thio-/Selenoreactive Elements

High exposures to soft electrophiles have the potential to incapacitate various sulfur- and Se-dependent metabolic processes, thus disrupting many redox regulatory mechanisms that are required for healthy cell growth and function, particularly in brain and endocrine tissues. Increased selenium status is known to counteract the adverse effects of elevated exposures to neurotoxic electrophiles such as Hg, cadmium (Cd), lead (Pb), and vanadium (V) [139]. These electrophilic elements may all be capable of selective, irreversible inhibition of selenoenzyme activities similar to the mechanism of Hg toxicity [140, 141]. However, relationships between Se status and the neurotoxic effects of these other elements have not been adequately examined, and additional mechanisms of toxicity have been recognized for some of these elements [142]. Because it is rich in cysteine, hair has often been used to provide a reflection of circulating amounts of thio-reactive electrophiles present in exposed individuals. The concentrations of Hg, As, Cd, or Pb in hair do not indicate consistent relationships with ASD incidence; however, some studies report unusually low concentrations in hair samples and suggest that individuals with ASD have diminished abilities to eliminate toxic metals [143, 144]. This is consistent with the finding that lower Hg concentrations are present in the hair of young (<6 y) children with ASD [144–146] although Majewska et al. [146] found older ASD children have higher hair Hg. Although serum was studied instead of RBC's or whole blood, a large study of Hg in relation to autism [147] reported finding no significant differences between nonautistic and ASD children. This is supported by the finding that no distinctions in expression levels of four genes that are known to respond to metal exposures were noted between ASD and typical children [148]. However, Stamova et al. [149] found a distinctive correlation between gene expression and blood Hg levels in boys with autism suggesting it is associated with a different pattern of gene transcription in response to Hg exposure. The ability of both Hg and Se to exert epigenetic effects was recently demonstrated in embryonic stem cells [150]. Several studies have reported potent toxic effects of methylmercury on neural stem cell differentiation and survival [151, 152], indicating its potential capacity for altering gene expression during development.

To prevent bacterial contamination in multiple-dose vials, thimerosal, the ethylmercury derivative of thiosalicylic acid, has been used as a preservative in various medical products, including vaccines. As autism rates increased, Bernard et al. [153] suggested that vaccine-derived Hg might be a contributing cause, a highly controversial proposal [154–156]. As a result, thimerosal was removed from all pediatric vaccines, except for some influenza vaccines, in the United States starting in 2001, but the incidence of autism continued to rise [157], furthering the doubts that vaccine-derived Hg exposures contributes to autism incidence. While a number of epidemiological studies do not indicate an association between thimerosal exposure and ASD [158, 159], possible associations between developmental disorders with Hg-containing vaccines [157] and delayed or even transgenerational influence of epigenetic changes have been suggested [160]. Such genetic or epigenetic defects of the antioxidant enzyme system could cooperatively interact with other environmental electrophiles and make vulnerable individuals more sensitive to exposure levels that would otherwise be harmless. Since deficits in selenoenzyme synthesis or function can increase the potential for oxidative stress and epigenetic dysregulation, sensitivity to neurotoxins, such as Hg and similar soft electrophiles, may differ among individuals. In this regard, there have been several reports of decreased GPx activity in autism [161, 162] and selective transmission of GPx-1 allelic variants [121]. Interestingly, Paşca et al. [120] found an inverse correlation between homocysteine and GPx activity in autistic subjects, indicating an association between low GPx activity and impaired methylation. Therefore, Castañeda et al. suggested that the removal of thimerosal from vaccines might not be immediately reflected as a reversal of epigenetic effects, especially if they involved effects on germline cells [163]. Notably, lower levels of DNA methylation increase novel insertions of transposable elements and increase the frequency of CNVs in germline cells [164–166], which are also elevated in autism. Therefore, the effects of elemental electrophiles on selenometabolism may be a contributing factor to ASD etiology and/or progression in vulnerable individuals.

### 6.2. Thio-/Selenoreactive Organic Electrophiles

Organic molecules such as acrylamide, acrolein, and diethyldithiocarbamate are structurally diverse, but these electrophilic species all share the potential to chemically react with strong nucleophiles [167]. Just as for inorganic electrophiles, the most likely target will be the most nucleophilic moieties of enzymes, such as Sec or thiols of Cys residues. Acetaminophen, the most commonly used analgesic and antipyretic drug in much of the world, is associated with toxic effects at high exposures. Excessive intake leads to impaired sulfur metabolism and life-threatening hepatotoxicity, involving depletion of GSH [168]. A survey of parents reported a higher frequency of acetaminophen use after the MMR (measles-mumps-rubella) vaccine for autistic children than for unaffected children [169], leading to the suggestion that use of acetaminophen might be causally linked to an increase in autism rates [170].

Interestingly, the major acetaminophen-binding protein in the liver is Se binding protein-2 (SeBP2) [171, 172]. Both SeBP1 and SeBP2 bind Se, but not in the Sec form characteristic of the genetically encoded selenoproteins. Increased expression of SeBP2 is associated with increased susceptibility to acetaminophen cytotoxicity [173]. In view of the male predominance of autism, it is interesting to note that SeBP2 levels are higher in males [174] and their vulnerability to acetaminophen hepatotoxicity is also greater. Males also display a decreased capacity to restore their GSH levels to normal [175] following high acetaminophen exposures.

N-acetylcysteine protects against acetaminophen-induced hepatotoxicity by maintaining or restoring hepatic concentrations of GSH [176]. Glutathione is required to inactivate *N-*acetyl-*p-*benzoquinone imine (NAPQI), a metabolic product of acetaminophen breakdown that is thought to be the proximal cause of hepatotoxicity following acetaminophen overdoses. When excessive quantities of NAPQI are formed, the primary metabolic (glucuronide and sulfate conjugation) pathways apparently become saturated. N-acetylcysteine is thought to counteract toxicity by either reducing NAPQI to the parent compound or providing sulfhydryl for conjugation of this metabolite [176]. If supplementation with a sulfhydryl-containing compound such as N-acetylcysteine can directly inactivate NAPQI, supplementation with Se to restore selenoenzyme status may be an important adjunct therapy to restore healthy redox status in the affected tissues. The significance of the liver in providing Se for delivery to the brain suggests that compromised Se availability in the liver temporarily induced by exposures to NAPQI and/or other electrophiles could diminish the amount of Se the brain receives. Exposures to agents which could cause either prolonged or excessive diminishments in the supply of Se to neuroendocrine tissues may therefore be important factors to consider in relation to ASD.

## 7. Summary and Conclusions

The nosology of ASD is complicated by the difficulties to differentiate the syndrome into subsets with similar symptoms and distinct etiologies. Children that share the diagnosis of ASD represent more than one distinct pathophysiological condition. Recognizing and distinguishing between groups with separate etiologies require identification of objective laboratory indices that clinicians can use for diagnosis and to monitor progression and treatment effects. The objective of this review is to discuss metabolic defects that may contribute to the onset and pathology of ASD, particularly in relation to Se physiology.

**Figure 3:**
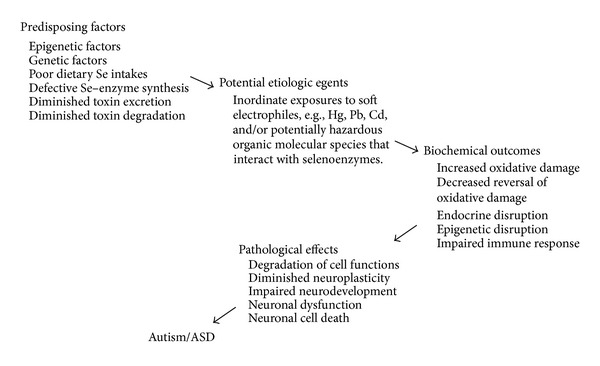
Depiction of potential etiologic contributors to disruptions of selenoenzyme physiology that may lead to disruptions of redox control and pathological consequences of autism and ASD. The factors and agents depicted are not all necessarily involved, but increases in predisposing factors along with additive contributions of increased exposures to thio- and selenoreactive electrophiles would be expected to increase the likelihood of progression to pathology.

Existing evidence indicates children with ASD have disruptions in GSH metabolism, and that impaired selenoenzyme and thiol metabolic pathways may be involved. These disruptions could occur as the result of multiple exposures to elemental and organic electrophiles (Figure 3) with exacerbations related to congenital vulnerabilities. Therefore, genetic predisposing factors for ASD may be exacerbated secondary to high aggregate exposures to electrophiles acquired through environmental, pharmaceutical, or foodborne routes. Currently, no specific causal agents or conditions have been recognized in ASD and not all children with potentially predisposing congenital defects or environmental exposures acquire ASD. This may indicate that the contributing effects of causal agents have low potency or that more than one predisposing factor is required. Therefore, a confluence of determinants of individual vulnerabilities and sufficiently high exposures to potential causal agents may be required to result in pathological effects. Likewise, the mechanisms of action may additively or synergistically accentuate risks to individuals with underlying genetic, epigenetic, or nutritional susceptibilities.

Based upon the mechanisms outlined above, interventions designed to diminish oxidative damage and support methylation capacity could improve the health of individuals afflicted with ASD, particularly those with inadequate antioxidant defenses. In such individuals, dietary interventions may offer low-risk approaches with potential for significant improvements in neurodevelopmental outcomes. Although the etiology and pathology of ASD remain poorly resolved, research suggests that afflicted children may benefit from treatments designed to improve their antioxidant capacity.

## Figures and Tables

**Table 1:** Glutathione and oxidative stress in autism.

Authors (Reference)	Control *n*	Autistic *n*	GSH status	Additional related findings
James et al. [14]	33	20	46%↓	↓GSH/GSSG, ↓SAM/SAH, ↓cysteine
James et al. [15]	73	80	32%↓	↓GSH/GSSG, ↓SAM/SAH, ↓cysteine
D. A. Geier and M. R. Geier [16]	Lab-based normal values	10	36%↓	↓Cysteine
Adams et al. [17]	55	43	21%↓	↓SAM, ↓cysteine, ↓vitamin E, ↑FIGLU
Paşca et al. [18]	13	15	33%↓	↓Cysteine
Pastural et al. [19]	12	15	35%↓	↓Cysteine
Al-Gadani et al. [20]	30	30	27%↓	↑Lipid peroxides, ↓vitamin E, ↓SOD, ↓GPx
Melnyk et al. [21]	40	40	29%↓	↓GSH/GSSG, ↓SAM/SAH, ↓cysteine, ↓DNA methylation
James et al. [22]	42	40	28%↓	↓GSH/GSSG, ↓SAM/SAH, ↓cysteine
Geier et al. [23]	120	28	24%↓	↓Cysteine
Geier et al. [24]	Lab-based normal values	28	24%↓	↑GSSG, ↓cysteine, ↓taurine, ↓sulfate

**Table 2:** 

Selenoprotein	Functions	References
GPx1	Detoxifies peroxides in aqueous compartment of cellular cytosol	[58]
GPx2	Expressed in cytosol of liver and tissues of the digestive system	[59]
GPx3	Synthesized primarily by kidney; secreted into plasma for transport to other tissues	[60]
GPx4	Prevents and reverses oxidative damage to lipids in brain and other tissues	[61]
TRx1	Reduces T(SH)_2_, vitamin C, polyphenols, and other substrates to regulate intercellular redox state	[62–64]
TRx2	Located in mitochondria and controls and regulates redox state	[63, 64]
TRx3	Reduces mitochondrial glutathione disulfide, abundant in testes	[63, 64]
MsrB1	Restores oxidatively damaged methionine (R-sulfoxides) to native configuration	[64]
DIO1	Converts T_4_ (thyroxine) prohormone into T_3_ (active thyroid hormone)	[65]
DIO2	Regulates thyroid hormone status, activating as well as inactivating T_3_	[65]
DIO3	Activates thyroid hormone in brain, placenta, important in fetal development	[65]
SPS2	Creates the Se-phosphate precursor required for synthesis of all selenoproteins	[64]
SelM	Notably high expression levels in the brain, possible thiol-disulfide oxidoreductase	[64, 66]
SelN	Interacts with ryanodine receptor, mutations result in congenital muscular dystrophy	[64]
SelP	Transports Se in plasma (10 Sec/molecule) and delivers Se to brain and endocrine tissues	[64]
SelW	Expressed in a variety of tissues and may regulate redox state of 14-3-3 proteins	[64, 66, 67]
Sel15	Oxidoreductase that may assist in disulfide formation and protein folding	[64]
